# Adaptability of Older Adults at the Onset of the COVID-19 Pandemic

**DOI:** 10.1093/geroni/igab046.2720

**Published:** 2021-12-17

**Authors:** Laurie Blackman, Donna Wang, Kathryn Krase, Joyce Roberson-Steele, Annette Clarke-Jones, Latoya Attis

**Affiliations:** 1 Yeshiva University, Wurzweiler School of Social Work, Silver Spring, Maryland, United States; 2 Springfield College, Springfield College/Springfield, Massachusetts, United States; 3 Yeshiva University, Yeshiva University/New York, New York, United States

## Abstract

It is important to understand the unique experiences and perspectives of older adults who were required to incorporate critical adjustments to behavior during the onset of the COVID-19 pandemic. An anonymous, cross sectional survey was administered online through Qualtrics Survey Software in June 2020. The results of this study found that older adults utilized different sources of information than younger adults; they were more likely to read the newspaper or listen to the radio, and less likely to rely on social media for information. Older respondents in this study reported coping with the COVID-19 outbreak better than younger respondents, were less likely to report that they were personally affected by the virus, and less likely to feel overwhelmed by the amount of information about COVID-19. The findings of this study highlight resilience in older adults not found in younger adults, and provide an important step in identifying policy and practice suggestions to reduce negative repercussions for older adults experiencing the current crisis, as well as future generations of older adults who might experience similar events.

